# Decoding cardiac homeostasis and injury: the evolving landscape of spatial transcriptomics

**DOI:** 10.3389/fcell.2026.1772507

**Published:** 2026-05-25

**Authors:** Anis Hanna

**Affiliations:** The Wilf Family Cardiovascular Research Institute, Department of Medicine (Cardiology), Albert Einstein College of Medicine, New York City, NY, United States

**Keywords:** heart failure, multi-omics, myocardial infarction, single cell, spatial transcriptomics

## Abstract

The heart is a structurally complex organ where function is intimately tied to the precise spatial organization of diverse cell types. While single-cell RNA sequencing (scRNA-seq) has revolutionized cardiovascular research by providing a high-resolution “parts list” of the heart, the requisite tissue dissociation destroys the critical spatial context of intercellular communication and microenvironmental niches. Spatial transcriptomics (ST) has emerged as a transformative technology that bridges this gap, enabling the mapping of gene expression back to its histological coordinates. This review discusses the rapidly evolving landscape of spatial technologies, categorizing them into sequencing-based methods (e.g., Visium, Stereo-seq) which offer transcriptome-wide discovery, and imaging-based methods (e.g., MERFISH, Xenium, CosMx) which provide subcellular resolution with high sensitivity. We highlight recent applications of these tools in uncovering the spatial architecture of the heart at homeostasis and following injury. Furthermore, we explore the next frontier of spatial multi-omics, including simultaneous profiling of the proteome via sequential immunofluorescence (seq-IF) and expansion proteomics (iPEX), as well as chromatin accessibility. We conclude by discussing the computational challenges and future perspectives of integrating these multi-modal datasets to construct comprehensive atlases of the healthy and injured heart.

## 1 Introduction

Spatiotemporal coordination of diverse cell lineages maintains cardiac homeostasis and orchestrates the profound structural and functional transformations during cardiac remodeling. For decades, our understanding of these cell-specific homeostatic and reparative mechanisms was constrained by the resolution limits of genomic technologies. Bulk RNA sequencing, while powerful, averages gene expression across cell subpopulations, effectively obscuring the logic that governs cellular heterogeneity and phenotypic transitions. The development of single-cell RNA sequencing (scRNA-seq) altered the field by cataloging the “parts list” of the heart, identifying distinct cardiomyocyte, stromal and immune cell diversities. Yet, scRNA-seq inherently requires tissue dissociation, a process that strips cells of their physiological context and severs the mechanical and chemical cues provided by the extracellular matrix. The advent and rapid maturation of spatial transcriptomics (ST) may soon overcome these limitations. ST preserves the architectural context of gene expression, enabling mapping of the transcriptome back to the histological landscape with ever-increasing resolution. This review briefly explores the application of these cutting-edge tools to cardiac biology. The review synthesizes evidence from systematic benchmarking of spatial transcriptomics platforms, explores the development of open-source, cost-effective workflows, and examines how to leverage ST to study microenvironments of cardiac homeostasis and disease.

### 1.1 The spatial transcriptomics ecosystem: platforms and performance

**FIGURE 1:**
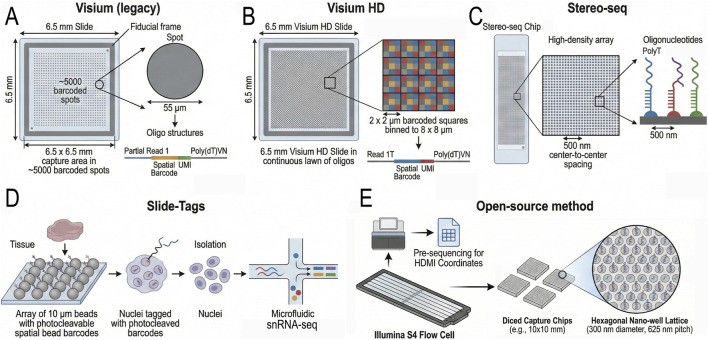
Overview of Sequencing-Based Spatial Transcriptomics (sST) Platforms. **(A)** The legacy Visium platform utilizes capture spots with a diameter of 55 µm. This resolution aggregates the transcriptomes of 2–10 cells, effectively obscuring cellular heterogeneity and necessitating computational deconvolution to infer cellular composition. **(B)** Visium HD employs 2 µm resolution bins to significantly improve spatial granularity, providing single-cell resolution across the whole transcriptome. **(C)** Stereo-seq utilizes DNA nanoball (DNB) technology on lithographically etched chips to achieve a spot size of 220 nm and a pitch of 500–715 nm. This nanoscale resolution enables the capture of transcripts with high spatial fidelity across large fields of view. **(D)** Slide-tags employs a strategy of spatial indexing where nuclei are tagged with photocleavable barcodes from an array of 10 µm beads prior to isolation. This method reconstructs spatial information computationally and is compatible with standard single-nucleus RNA sequencing workflows. **(E)** Open-source methods, such as Seq-Scope or Nova-ST, repurpose Illumina sequencing flow cells as ultra-high-density spatial arrays. By performing solid-phase amplification of randomly barcoded oligonucleotides directly on the flow cell surface, these methods generate RNA-capturing clusters spaced approximately 0.5 µm apart.

The landscape of spatial transcriptomics has evolved from low-resolution arrays to platforms capable of subcellular and single-molecule precision. Selecting the appropriate technology requires a nuanced understanding of trade-offs between resolution, effective capture area, gene throughput, sensitivity, technical feasibility, and cost (You et al., 2024; Wang H. et al., 2025). While this is not an exhaustive classification of all available spatial transcriptomics methods, the current ecosystem can be broadly categorized into (Figure 1, Table 1) sequencing-based (sST) and imaging-based (iST) methodologies.

**TABLE 1:** Comparison of spatial transcriptomics platforms.

Platform (type)	Resoluti-on	Capture area	Tissue	UMI/Probebased	Gene coverage	Sensitivity (UMIs or Transcripts)*	Key strengths	Key limitations
Visium V1/V2 (Ståhl et al., 2016; Du et al., 2025; Hunter et al., 2021) (sST)	55 µm spots (aggregates 2–10 cells)	6.5 × 6.5 mm	FFPE/Fresh Frozen	UMI-based	Whole Transcriptome	∼5,000 - ∼30,000 UMIs per spot (multicellular)	Useful for broad tissue mapping; established workflow for general architectural overview Widely adopted	Low resolution (multicellular); averaging of transcriptomes within spots which requires deconvolution
Visium HD (Oliveira et al., 2025; Ren et al., 2025) (sST)	2–8 µm bins (Subcellular/Single-cell)	6.5 × 6.5 mm	FFPE/Fresh Frozen	UMI-based	Whole Transcriptome	∼200–600 UMIs per 8 µm bin	Continuous high-resolution grid; better diffusion control than other array methods	High cost Bin-based analysis may require aggregation for low-expressed genes
Stereo-seq V1^27^ (sST)	∼220 nm spots/0.5 µm bins	Up to 13 × 13 cm	Fresh Frozen	UMI-based	Whole Transcriptome	∼200–400 UMIs per 8 µm bin	High-resolution; scalable to centimeters	High cost Lateral diffusion of transcripts observed in some tissues
Stereo-seq V2^7^ (sST)	∼220 nm spots/0.5 µm bins	Up to 13 × 13 cm	FFPE/Fresh Frozen	UMI-based	Whole Transcriptome	Comparable or higher sensitivity than Stereo-seq V1	Optimized for FFPE samples; random priming provides uniform gene body coverage	High cost. Requires sophisticated segmentation; data sparsity at raw resolution
Slide-seqV2 (Stickels et al., 2020; Rodriques et al., 2019) (sST)	10 µm beads	∼3 × 3 mm to 7 mm^2^	Fresh Frozen	UMI-based	Whole Transcriptome	400–500 UMIs per bead	Near single-cell resolution; cost-effective for small regions	Small capture area; random bead array requires registration. Performance and cell recovery rates varies by tissue
Slide-tags (Curio Trekker; sST) (Russell et al., 2023)	Single nucleus	6 mm × 6 mm and10 mm × 10 mm	Fresh Frozen	UMI-based	Whole Transcriptome	Comparable to snRNA-seq, albeit with nuclei recovery rate of ∼25%	Offers single-cell resolution by physically tagging nuclei before being processed as snRNA-seq	Loss of tissue morphology (cells are disassociated). Cytoplasmic RNA is lost. Nuclei recovery rate of ∼25%
Flow Cell-Based Methods (Kim et al., 2024; Poovathingal et al., 2024; Schott et al., 2024) (sST)	Sub-micrometer (∼0.5 µm)	10 × 8 mm and Scalable (Flow cell dependent)	Fresh Frozen	UMI-based	Whole Transcriptome	800–2,000 UMIs per cell	Low-cost subcellular resolution	Requires technical expertise to repurpose sequencing flow cells. Requires custom wet-lab setup
Xenium (iST) (Ren et al., 2025)	Subcellular/Single-molecule	12 × 24 mm	FFPE/Fresh Frozen	Probe-based	Targeted (300–5,000+ genes)	300–600 counts per 8 µm bin	Superior specificity (low background noise); high sensitivity; reliable cell segmentation	Limited to pre-defined gene panels Requires expensive, specialized instrumentation
CosMx (iST) (Ren et al., 2025)	Subcellular/Single-molecule	15 × 20 mm	FFPE/Fresh Frozen	Probe-based	Targeted (1,000–6,000+ genes)	250–600 counts per 8 µm bin	High plex capability (6K + genes); flexible capture area selection	Higher background noise/false positives compared to Xenium; longer run times for high plex
MERSCOPE (Wang et al., 2025a) (iST)	Subcellular/Single-molecule	1–3 cm^2^	FFPE/Fresh Frozen	Probe-based	Targeted (∼500–1,000 genes)	Lower transcripts count per cell compared to other iST methods	High localization accuracy; proven MERFISH chemistry	Lower plex compared to other iST methods; sensitivity drops significantly with lower quality RNA.

* Unique Molecular Identifier (UMI) and transcript counts vary by tissue type and depend on tissue quality, preparation and the technical variability within the spatial transcriptomics protocols.

#### 1.1.1 Sequencing-based spatial transcriptomics (sST)

Sequencing-based ST methods capture mRNA from tissue sections onto spatially barcoded arrays, which are then sequenced using next-generation sequencing. These platforms offer unbiased, whole-transcriptome coverage, making them ideal for discovery-driven research where target pathways may be unknown.

##### 1.1.1.1 The evolution from micro-to nano-patterned arrays

One of the most widely used sST methods is the Visium platforms (Ståhl et al., 2016) (V1 and V2) (Figures 1A,B), which utilize capture spots with a diameter of 55 µm. While revolutionary, this resolution aggregates the transcriptomes of 2–10 cells, necessitating computational deconvolution to infer cellular composition (Hanna et al., 2021). Recent innovations have pushed sST into the single-cell and subcellular scale. The Visium HD platform (Oliveira et al., 2025) employs 2 µm bins to provide whole transcriptome profiling on a single-cell resolution. While this significantly improves spatial granularity compared to the original 55 µm spots, the platform comes with a high cost. Another recent advancement is Stereo-seq (Chen et al., 2022) (Spatial Enhanced Resolution Omics-sequencing), which utilizes DNA nanoball technology on lithographically etched chips to achieve a spot size of 220 nm and a pitch of 500–715 nm (Figure 1C). This nanoscale resolution enables the capture of transcripts with high spatial fidelity across scalable fields of view (up to 13 × 13 cm). Benchmarking suggests that while Stereo-seq provides high resolution, it may suffer from greater transcript diffusion compared to other platforms, potentially blurring the boundaries between adjacent cells (You et al., 2024). Recently, Stereo-seq V2 was introduced to address the limitations of V1 (Zhao et al., 2025). By utilizing random primers rather than poly(A) capture, Stereo-seq V2 enables high-sensitivity profiling in FFPE or fresh frozen tissue sections.

Significant challenges remain for sST platforms. First, despite being unbiased, these methods are technically limited by capture efficiency, leading to gene dropout and data sparsity that complicate the detection of low-abundance transcripts. Second, lateral diffusion of mRNA during permeabilization can cause signal bleed-through, blurring spatial precision. Finally, accurate cell segmentation remains a computational challenge. Approaches relying on nuclear H&E stains may fail to distinguish the irregular, interdigitated boundaries of cells, leading to “contamination” or mixing of transcriptomes between adjacent cells. However, segmentation based on immunofluorescence of cell surface markers may be a plausible solution to this issue.

##### 1.1.1.2 Spatial transcriptomics using DNA-barcoded beads

Slide-seq (V1 and V2) (Stickels et al., 2020; Rodriques et al., 2019) represent sequencing-based spatial transcriptomics platforms that transfer tissue sections onto arrays of DNA-barcoded beads deposited on glass slides. In Slide-seq, 10-µm beads are arranged in a monolayer, enabling spatial mapping at cellular scale. Key downsides of Slide-seq include its small capture area, and its tissue-dependent performance (You et al., 2024). Slide-tags (Russell et al., 2023) (commercially available as Curio Trekker, Takara Bio), a related method, uses spatial indexing during tissue cryosectioning via combinatorial barcoding, followed (Figure 1D) by nuclei isolation, and snRNA-seq of the barcoded nuclei. While Slide-tags lacks in-section spatial coordinates, it reconstructs the spatial location of the dissociated nuclei computationally. A key limitation of these barcoded bead methods is the partial recovery of cells; Slide-tags only recovers ∼25% of nuclei due to imperfect bead-tissue contact and nuclei isolation efficacy. Additionally, snRNA-seq-related limitations are unavoidable with Slide-tags, including doublets, and since only nuclei are analyzed, cytoplasmic RNA is by default lost.

##### 1.1.1.3 Repurposing sequencing flow cells for an affordable alternative

A major barrier to the widespread adoption of high-resolution sST has been the prohibitive cost and limited accessibility of commercial arrays. A novel class of open-source methods (Kim et al., 2024; Poovathingal et al., 2024; Schott et al., 2024) has emerged that repurposes Illumina sequencing flow cells as ultra-high-density spatial arrays (Figure 1E). By performing solid-phase amplification of randomly barcoded oligonucleotides directly on the flow cell surface, these methods generate RNA-capturing clusters spaced approximately 0.5–0.8 µm apart. This sub-micrometer resolution allows for the visualization of subcellular architectures, such as the distinction between nuclear and cytoplasmic transcript pools. This innovative repurposing of a commonly used platform reduces the cost per sample to a fraction of commercial equivalents, democratizing access to high-resolution spatial profiling and enabling large-scale atlasing. However, the primary limitation of these workflows is the level of technical expertise required to ‘hack’ the flow cell, through lengthy workflows that may not be feasible outside genomics core facilities. In addition, the active spatial capture area depends on the design of the flow cell, limiting the size of the chip to ∼8 mm^2 12^, which restricts its utility for large tissue sections.

#### 1.1.2 Imaging-based spatial transcriptomics (iST)

##### 1.1.2.1 Principles of common iST methods

Imaging-based ST methods, including Xenium (Janesick et al., 2023) (10x Genomics), CosMx (He et al., 2022) (NanoString), and MERSCOPE (Chen et al., 2015; Moffitt et al.) (Vizgen), rely on cyclic fluorescence *in situ* hybridization (FISH) or *in situ* sequencing chemistry (Table 1). These platforms offer single-molecule precision and subcellular resolution but are typically limited to targeted gene panels rather than the whole transcriptome. In addition, the imaging-based detection in these iST methods usually requires specialized expensive integrated workstations. Xenium employs circularizable padlock probes with rolling circle amplification to generate discrete, bright signals, achieving the highest sensitivity per gene across matched samples. Similarly, CosMx utilizes branched chain hybridization for signal amplification. Crucially, these amplification strategies render both platforms robust to RNA degradation, ensuring consistent performance even in standard archival FFPE tissues. MERSCOPE, on the other hand, relies on direct probe hybridization (MERFISH chemistry) without enzymatic amplification; while this tiling approach yields high sensitivity in high-quality samples, it renders the platform highly vulnerable to RNA fragmentation. Consequently, MERSCOPE exhibits significantly variable performance in FFPE tissues.

In addition to the challenges with cell segmentation, a technical challenge unique to all iST methods is “optical crowding.” In regions of extremely high transcript density, fluorescent signals can overlap, exceeding the optical diffraction limit and hindering accurate spot decoding.

##### 1.1.2.2 Multiplexing capacity and cellular resolution of common iST methods

The gap between targeted validation and broad discovery is rapidly closing. Xenium and CosMx now utilize expanded panels targeting 5,000 and 6,000 genes, respectively. In addition, Xenium also supports custom add-on panels, allowing the inclusion of targets of interest to validate mechanisms identified in scRNA-seq discovery studies. CosMx leverages this massive multiplexing capability to detect the highest absolute number of unique genes and total transcripts per cell. While earlier CosMx protocols exhibited higher background noise and lower gene-wise correlation with single-cell data, updated chemistry has markedly improved sensitivity and FDR to approach the performance of Xenium (Wang H. et al., 2025). CosMx demonstrates comparable segmentation precision to Xenium, whereas MERSCOPE has shown lower segmentation accuracy and a higher propensity for identifying cells with co-expression of disjoint markers, reflecting the challenges of non-amplified detection in complex tissue environments (Wang H. et al., 2025).

### 1.2 Computational deconvolution: resolving the cellular mesh

Despite the advent of subcellular platforms, the majority of currently available ST datasets are based on the legacy Visium platforms. Resolving the cellular composition of a 55 µm spot is non-trivial. Computational deconvolution methods are therefore essential to infer the spatial distribution of cell types. Deconvolution algorithms generally integrate spatial transcriptomics with reference scRNA-seq data to estimate cell-type proportions. Tools such as Cell2location (Kleshchevnikov et al., 2022) and RCTD (Robust Cell Type Decomposition) (Cable et al., 2021) utilize probabilistic modeling to map single-cell signatures onto spatial spots. Cell2location employs a negative binomial regression model that accounts for technical variability and is particularly effective at estimating absolute cell abundance. RCTD uses a Poisson-based framework to robustly decompose spots containing mixtures of cells. Other reference-based methods offer distinct advantages; for instance, CARD (Conditional Auto-Regressive-based Deconvolution) (Ma and Zhou, 2022) leverages spatial correlation information between spots to improve cell-type inference, while SPOTlight (Elosua-Bayes et al., 2021) utilizes a seeded Non-negative Matrix Factorization (NMF) regression, initialized with single-cell marker genes, to deconvolute mixtures.

The reliance on external atlases for ST deconvolution can introduce biases and may not capture cell types unique to certain conditions or species. In scenarios where a matched single-cell atlas is unavailable, for example, in rare congenital defects or specific pathological states, reference-free methods are essential. SpatialDeX (Liu et al., 2025), for example, uses a regression model to estimate cell-type proportions and identify spatial domains without external single-cell references.

Recent advancements in multimodal integration utilize deep learning to enhance transcriptomic deconvolution with high-resolution H&E imagery. Frameworks like Thor (Zhang et al., 2025) and MISO (Schmauch et al., 2025) extract quantitative morphological features including nuclear density, size, and orientation to refine spot-level data and constrain cell-type mapping. This is particularly relevant for the heart, where the distinct morphology of cardiomyocytes vs. interstitial cells can guide the assignment of transcripts.

### 1.3 Spatial mapping to decode cardiac homeostasis and injury

#### 1.3.1 Specialized cellular niches of the normal human heart

Establishing a high-resolution spatial baseline of the healthy human heart is an essential prerequisite for deciphering the complex structural changes that drive cardiac disease. Kanemaru et al. utilized spatial transcriptomics to delineate the precise microanatomy of human cardiac niches, demonstrating that the cardiac conduction system is not purely cell-autonomous (Kanemaru et al., 2023). Analysis of Visium ST and inference of cell-cell communications showed that pacemaker cells within the sinoatrial node colocalize with a specialized glial population supporting glutamatergic signaling, and they identified an epicardial immune niche enriched with plasma B cells acting as a localized protective barrier. Furthermore, they achieved the first transcriptome-wide spatial profiling of the intrinsic cardiac nervous system. While this study provides a comprehensive atlas of homeostatic human cardiac architecture, the study heavily relied on cell2location algorithm to computationally infer cellular composition from static external references, given the limited Visium resolution. Consequently, the reported findings of active glutamatergic signaling between pacemaker cells and glia represent statistically inferred transcriptomic probabilities within a shared micro-neighborhood.

#### 1.3.2 The spatially distinct remodeling niches in the ischemic and failing heart

Myocardial infarction (MI) induces a profound spatial reorganization of the heart, creating distinct zones that can be roughly divided into an infarct, infarct border, and remote remodeling myocardium (Hanna et al., 2021; Hanna et al., 2020; Hanna and Frangogiannis, 2019; Prabhu and Frangogiannis, 2016). One of the early uses of Visium to spatially map the human infarcted heart identified distinct cell niches that characterize “myogenic”, “ischemic”, and “fibrotic” tissue areas (Kuppe et al., 2022). By integrating single cell multi-omics with spatial data, Kuppe et al. identified a fibro-myeloid signaling axis where inflammatory niches, enriched with SPP1+ macrophages, are geographically surrounded by fibrotic-rich niches. This interaction is mediated by stage-specific signaling shifts: PDGF-C, PDGF-D, and THBS1 characterize the ischemic phase, while ADAM17 and TGFB1 drive crosstalk during fibrotic remodeling. Critically, the low-resolution Visium used in the study necessitated deconvolution, meaning that the proposed cellular transitions are based on computational inferences.

In a murine model of MI, Yamada et al. proposed that the infarct border zone is not merely a passive site of tissue transition, but an active, mechanosensitive niche. Using spatial transcriptomics, they revealed that local mechanical stretch induces the spatially restricted expression of *Csrp3* and other Z-disc-related genes, which may serve as adaptive regulators to mitigate adverse remodeling. However, while the study establishes *Csrp3* as an adaptive regulator, its reliance on Visium ST limits the ability to prove whether these stretch responses are purely cell-autonomous or depend on direct contact with neighboring fibroblasts or immune cells (Yamada et al., 2022). In a similar model, Calcagno et al. demonstrated that the infarct border zone is driven by mechanical destabilization rather than ischemia alone. Using spatial transcriptomics, they identified distinct border zone cardiomyocyte layers expressing *Nppa* and *Xirp2* that physically align with morphological distortion and myofibroblast activity. This led to their loss of neighbor hypothesis, suggesting that localized cell death mechanically stretches surviving cells. Critically, while this mechanical framework is innovative, definitively uncoupling localized ischemia from physical stretch *in vivo* remains challenging (Calcagno et al., 2022).

Building on this architecture, Ninh et al. attempted to redefine sterile inflammation post-infarction. They revealed that mechanical stress in the border zone causes cardiomyocyte nuclear rupture and DNA leakage, triggering a localized type I interferon response. These findings propose a model of post-infarction inflammation that moves beyond being exclusively myeloid-cell-driven to being actively initiated by mechanically stressed cardiomyocytes. Critically, while the study elegantly links mechanical strain to localized immunity, its reliance on static spatial snapshots makes it difficult to determine whether these interferon-induced clusters represent a transient protective mechanism or a sustained maladaptive response (Ninh et al., 2024).

Overall, despite serving as an important early spatial resource for the chaotic environment of the infarcted heart, the low resolution of Visium used in these studies makes it difficult to attribute spatially resolved transcriptional changes to a specific cell phenotype or state.

Distinct from the other spatial transcriptomics methods discussed above, GeoMx Digital Spatial Profiler enables high-plex profiling of user-defined Regions of Interest (ROIs). Lee et al. (2025) utilized GeoMx to analyze cardiac tissue segments with cardiomyopathies, proposing heart failure as a mosaic of localized microenvironmental failures. In cardiomyocytes, specific morphological disarray, such as hypertrophic myocyte disorganization, was linked to localized transcriptional suppression of ribosomal proteins. On the other hand, fibrotic regions independently harbored pro-inflammatory endothelial signatures that were linked to leukocyte transmigration. Critically, however, because GeoMx relies on user-defined ROIs, this approach introduces inherent selection bias. By exclusively profiling predefined pathological structures, the study potentially overlooks the early transitional tissue states.

#### 1.3.3 Microanatomical profiling of cardiac granulomas and myocarditic cardiac injury

Liu et al. (2022) studied the immune architecture of cardiac sarcoidosis, demonstrating that granulomas compartmentalize specific macrophage differentiation networks and antigen presentation pathways distinct from generalized inflammation. By utilizing the GeoMx platform, the authors mapped localized upregulation of JAK-STAT and Toll-like receptor signaling directly to these lesions. Critically, because this approach requires manual selection of morphologically obvious ROIs, it inherently overlooks the transcriptomic landscape of visually unaffected adjacent tissue. This limitation may prevent a complete mechanistic understanding of the early spatial triggers that initiate granuloma formation before overt structural remodeling occurs.

Utilizing Visium ST and Slide-seq to map the microanatomy of viral myocarditis in neonatal mice, Mantri et al. (2022) revealed that tissue injury and host response are highly spatially restricted to localized myocarditic micro-lesions showing an accumulation of *Cxcl9*-high endothelial cells and infiltrating immune cells. The study also proposed the immediate border zone around these lesions as a protective barrier that actively mounts spatially restricted interferon response that may help contain the virus. While spatial transcriptomics elegantly defined these immunological containment barriers, relying on static snapshots limits our ability to track the dynamic response of these localized barriers, and whether they successfully contain the infection or eventually fail and coalesce to cause macroscopic heart failure.

### 1.4 Beyond transcriptomics: spatial multi-omics

The rapidly evolving field of spatial biology may soon enable the integration of high-resolution spatial multi-omics. While transcriptomics infers cellular state, phenotype is ultimately dictated by the translation of these transcripts into functional proteins. In addition, to fully decode cellular identity, one should look upstream to the epigenome. Spatial ATAC-seq (Assay for Transposase-Accessible Chromatin) has recently emerged, enabling the mapping of chromatin accessibility in tissue context. DBiT-seq (Liu et al., 2020) (Deterministic Barcoding in Tissue) achieves spatial epigenomics by utilizing microfluidics to deliver unique spatial barcodes and Tn5 transpososis on top of tissue sections. Major limitations of this platform include the relatively wide gaps between microfluidic channels (dead space) effectively prohibiting continuous mapping of the adjacent cells.

For unbiased spatial proteomic discovery, iPEX (Wang F. et al., 2025) (*in situ* imaging Proteomics via Expansion) integrates hydrogel-based tissue expansion with MALDI mass spectrometry imaging. By isotropically expanding the tissue scaffold, iPEX overcomes the diffraction and sensitivity limits of mass spectrometry, enabling the detection of 600–1,500 proteins with a high spatial resolution (1–5 µm). Complementary to the unbiased approaches, sequential immunofluorescence (seq-IF) (Kuehl et al., 2025) could serve as a method of targeted spatial proteomics, by enabling high-plex protein imaging through iterative cycles of antibody staining, imaging, and antibody elution. Unlike other targeted spatial proteomics methods that require DNA- or metal-conjugated antibodies, seq-IF relies on iterative cycles of staining with standard, off-the-shelf primary antibodies. The core innovation of seq-IF is the use of a gentle antibody elution buffer that effectively strips the antibody complexes after imaging without significant antigen degradation or tissue damage.

## 2 Conclusion

The evolution of spatial biology is moving the field from a catalog of cell types to a functional understanding of cellular communities. By assigning spatial coordinates to transcriptomic and proteomic data, we may be able to interrogate how local microenvironments dictate cellular phenotypes and disease progression. As briefly discussed in this review, the ecosystem of spatial technologies is diversifying rapidly. Sequencing-based platforms are breaking the resolution barrier to approach subcellular precision, while imaging-based methods are expanding their plex capability to rival transcriptomic depth.

Looking forward, the integration of spatial multi-omics represents the next leap. The ability to simultaneously map cellular multiomes including gene expression, protein abundance, and chromatin accessibility will allow for the reconstruction of gene regulatory networks *in situ*. Realizing the full potential of these technologies requires overcoming significant hurdles in data integration, computational standardization, and cost. Despite these challenges, spatial biology is poised to become a valuable asset for mechanistic inquiry, offering unprecedented insights into the cellular sociology of the heart and paving the way for cellular subpopulation targeted therapies.
